# California

**Published:** 1897-09

**Authors:** 


### LEGISLATION.

*California.* This State has issued a little pamphlet giving the State pure-food law which passed in 1895; also the ordinance concerning contagious diseases, which is entitled "An ordinance concerning contagious or infectious diseases in animals and the sanitary conditions of dairies, slaughter-houses, markets and other places from which the food-supply is distributed; regulating the sale of cheese, butter, milk, and other products of cattle; providing for the inspection and branding of dairy cattle within the county of Santa Clara; the condemnation and killing of cattle affected with a contagious or infectious disease; prohibiting the disposal of cattle so condemned; prescribing certain duties of the veterinary inspector and health officer of that county, and providing for a punishment for a violation of its provisions." The details of this ordinance provide that the inspector is authorized to inspect all animals within the limits of the county of Santa Clara, and if necessary to condemn such animals if they are afflicted with any disease injurious to human life. After he has done so, he has the right of demanding those animals for destruction, and it shall be considered a misdemeanor for the owner to refuse in any way to accede to his request. The inspector also overlooks the dairies and all stock used for food-consumption. It is also necessary under this ordinance for all receptacles intended for the use of dairy products to be labelled concerning the contents, and also whether the dairy from which it came has been inspected or not. An ordinance entitled "An ordinance to regulate the private inspection of cattle with a view of ascertaining their pathological condition within the county of Santa Clara, State of California," is also included in this pamphlet, and relates to the inspection of cattle by other than the veterinary inspector. The tuberculin must be submitted to the veterinary inspector, however, before the same can be used. The inspection must be held with the knowledge of the inspector, and the notice must be in writing and served at least forty-eight hours before the day stipulated, and he is compelled to be present either in person or by deputy and to brand the cattle so inspected. He has the privilege of rejecting the inspection if he deems it advisable to do so. The ordinance creating the office of veterinary inspector and health officer, and prescribing his powers and duties, provides for the appointment of this officer, who holds his office at the pleasure of the supervisors. He is empowered to remove nuisances and to look after the inspection and sanitation of such affairs as come within his jurisdiction.

